# Isolation of Labile Multi-protein Complexes by *in vivo* Controlled Cellular Cross-Linking and Immuno-magnetic Affinity Chromatography

**DOI:** 10.3791/1855

**Published:** 2010-03-09

**Authors:** Stephanie A. Zlatic, Pearl V. Ryder, Gloria Salazar, Victor Faundez

**Affiliations:** Department of Cell Biology, Emory University; Department of Medicine, Division of Cardiology, Emory University

## Abstract

The dynamic nature of cellular machineries is frequently built on transient and/or weak protein associations. These low affinity interactions preclude stringent methods for the isolation and identification of protein networks around a protein of interest. The use of chemical crosslinkers allows the selective stabilization of labile interactions, thus bypassing biochemical limitations for purification. Here we present a protocol amenable for cells in culture that uses a homobifunctional crosslinker with a spacer arm of 12 Å, dithiobis-(succinimidyl proprionate) (DSP). DSP is cleaved by reduction of a disulphide bond present in the molecule. Cross-linking combined with immunoaffinity chromatography of proteins of interest with magnetic beads allows the isolation of protein complexes that otherwise would not withstand purification. This protocol is compatible with regular western blot techniques and it can be scaled up for protein identification by mass spectrometry^1^.

Stephanie A. Zlatic and Pearl V. Ryder contributed equally to this work.

**Figure:**
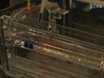


## Protocol

### 1. Preparing for Crosslinking

You will need to plate a sufficient number of cells to allow isolation of 500 μg of protein per standard tube reaction. A single tube may be enough for identification of putative interactors of a protein of interest by immunoblot. In this case bead bound material can be eluted with SDS-PAGE sample buffer (immunoprecipitation). For mass spectrometry analysis, the number of standard reactions should be increased at least ten times and protein complexes should be eluted by out-competition with a peptide antigen recognized by the antibody used (immunoaffinity chromatography). This strategy will allow the isolation of protein complexes free of immunoglobulin.
The optimal cell density for crosslinking is between 75% and 90% confluency. In high confluency conditions, cells tend to pile on each other, which decreases accessibility to the cell-permeable crosslinker to all cells.
Cell type and experimental condition should be labeled on the bottom of the cell plate. We routinely include controls with vehicle alone.
Prepare all solutions EXCEPT the DSP solution prior to crosslinking.
  Prepare a stock of phosphate-buffered saline buffer with CaCl_2_ 0.1 mM and CaCl_2_ 1 mM (PBS/Ca/Mg) prior to starting the experiment. The addition of calcium and magnesium ions is critical for adhesion of cells to the culture plate during the course of the experiment. Store at 4°C.
  50X Complete Protease Inhibitor Cocktail - 1 tablet dissolved in 1mL Milli-Q water. Store at -20°C.
  20% Triton X-100   Weigh out 10g Triton X -100 and dilute in total volume of 50 ml Milli-Q Water. Rock at 4°C overnight and store at 4°C until use. Do not store for more than 1 month. Use dilutions of this 20% stock to prepare the lysis and IP buffers described below.
  50X DSP Quenching Solution   1M TRIS to pH 7.4. Store at room temperature.
  Prepare a 10x Buffer A solution. 10X Buffer A:
    50 mL 1 M HEPES
    150 mL 5 M NaCl
    10 mL 0.5 M EGTA
    250 μL 2 M MgCl_2_
    Adjust pH to 7.4
    Bring final volume to 500 mL
    The 10X Buffer A solution is diluted to 1X Buffer A as needed.1x Buffer A is also used in lysate and immuno-magnetic-precipitation buffers. Buffer A 1X
    10 mM Hepes
    150 mM NaCl
    1 mM EGTA
    0.1 mM MgCl_2_
    pH 7.4
  Lysis Buffer   1X Buffer A + 0.5% Triton X-100.
  Immuno-Magnetic Precipitation Buffer (IP Buffer)   1X Buffer A + 0.1% Triton X-100.

### 2. Preparing the Crosslinking Solution

Prepare the DSP solution immediately before applying to cells. DSP is highly hydrophobic and should be dissolved in DMSO before diluting into PBS/Ca/Mg buffer.  Dissolve 40 mg of DSP in 1 mL of DMSO. This makes a 100 mM solution of DSP (The molecular weight of DSP is 404.42 g/mol).
Warm an appropriate volume of PBS/Ca/Mg to 37°C in order to facilitate dilution of DSP/DMSO into PBS. Volumes needed for assorted plate sizes:6-well plate   2 mL per well10 cm plate   10 mL per plate15 cm plate   20 mL per plate
Add 10μL of DSP/DMSO stock solution for every 1 mL of warm PBS/Ca/Mg. Add DSP/DMSO stock solution drop by drop with repeated mixing until all the DSP has dissolved. Make a control solution of 10 μL DMSO added to every 1 mL of PBS/Ca/Mg.
Place all PBS/Ca/Mg solutions in an ice-water bath no longer than 10 min.

### 3. Prepare Cells for Crosslinking

Prepare an ice-water bath that will fit all plates for crosslinking or vehicle control incubation.
Take plates from the 37°C incubator and place them immediately into the ice-water bath.
Wash cells two times with ice-cold PBS/Ca/Mg. Use the same volume that you will use for the crosslinker incubation (see section 2.2).
Remove second wash and add either vehicle control or DSP crosslinking buffer solution.
Incubate on ice for two hours. You should check the plates approximately every twenty minutes to ensure that all cells are covered by solution. You may notice a small amount of DSP precipitate out of solution. This is normal.

### 4. Inactivation of DSP Reaction

Prepare an 1X DSP quenching solution of 20 mM Tris pH 7.4 in PBS/Ca/Mg (20 μL of 1 M Tris pH 7.4 for every 1 mL of PBS/Ca/Mg).
Remove the vehicle control and crosslinking solutions.
Add ice-cold inactivation solution and incubate on ice for 15 minutes.

### 5. Cell Lysis

During DSP quenching incubation, prepare the cell lysis buffer of 0.5% Triton X-100 in Buffer A with Complete protease inhibitor cocktail.  Add 20 μL 50X stock solution of Complete for every 1 mL lysis buffer.
Following the 15 minute DSP quenching incubation, wash cells two times with PBS/Ca/Mg.
Add lysis buffer + Complete to cells and rock at 4°C for 30 minutes. Some suggested volumes of lysis buffer for assorted plate sizes:
  6-well plate   0.5 mL per well
  10 cm plate   1 mL per plate
  15 cm plate   1 mL per plate
After lysis, use a cell scraper to collect the cells from the plate. Be sure to keep lysates on ice to minimize protease activity
Spin cell lysates for 15 minutes at 4°C in a bench-top mini-centrifuge at top speed (15.000 x g).
Pipette the supernatant into a fresh tube. Discard the pellet.
Analyze protein levels and proceed with immunoprecipitation.

### 6. Preparing Immuno-magnetic Precipitation Beads

*Note: This step is usually started directly after beginning the 2 hour crosslinking incubation.*

Combine 30 μL of Dynal immunomagnetic beads, 500 μL of IP Buffer (1x Buffer A + 0.1% Triton X-100) and the appropriate amount of antigen-specific antibody to screwtop microcentrifuge tubes. Prepare antibody-free and non-specific antibody tubes for negative controls. The appropriate amount of antibody should be determined based on individual antigens during separate experiments. Label all IP tubes.
Allow the IP tubes to incubate in an end-over-end rocker at room temperature for two hours. Alternatively, incubate the IP tubes with antibody overnight at 4° the day prior to crosslinking.
After the incubation, do a quick 10 second mini-centrifugation to collect the beads at the bottom of the tube.
Slide the tubes into the magnetic holder, which will pull the magnetic beads away from the bottom of the tube. With the beads safely out of the way, you can now easily wash away any unbound antibody.
Use a small bore aspirator tip such as a gel loading tip, or p200 tip to remove the incubation buffer and any unbound antibody. As soon as the incubation volume is removed add in 1 mL of IP Buffer.
Cap the tube, gently re-suspend the beads, and incubate all IP tubes at room temperature for 5 minutes with end-over-end rotation.
Repeat the 5 minute wash step (steps 6.3 thru 6.6) once more with a fresh milliliter of IP Buffer.
The IP tubes can remain in the last wash incubation until the crosslinked lysate is ready to be added to the beads.

### 7. Incubate Crosslinked Lysate with Immuno-magnetic Beads

After the last wash spin immuno-magnetic beads, down in a mini-centrifuge for 10 seconds. Then proceed to slip the tubes into the magnetic holder.
Again, using a small bore aspiration tip, remove the incubation volume from the tube. Immediately add 500 μL of crosslinked cell lysate (assuming lysate at 1 μg/mL) to the tubes containing immuno-magnetic beads.  If you will be using peptide competition as a negative control, an appropriate amount of peptide should be included at this point. Previous experiments should be performed to determine appropriate peptide competition conditions.  Alternatively, you may want to have lysate free immuno-magnetic beads as a negative control. In this case, immediately add 500 μL of Lysate Buffer (1x Buffer A + 0.5% Triton X-100) + Complete to the immuno-magnetic beads.
Gently resuspend the beads. Resuspended beads should be incubated at 4° in an end-over-end rotation for 2 hours.

### 8. Wash Unbound Lysate from Beads

Once lysate has had sufficient incubation time, do a quick 10 second spin in a mini-centrifuge. Then slide the tubes into the magnetic holder. The magnetic holder should be kept in an ice bath for the remainder of the experiment.
Using a small bore aspirator tip, remove all unbound lysate. Immediately after all unbound lysate is removed add 1 mL of IP Buffer.
Cap the tube and gently resuspend the beads.
Once the beads are resuspended repeat the bead pelleting step (8.1).
Again aspirate off all of the incubation volume and immediately add 1 mL of IP Buffer, then cap the tube.
Gently resuspend the beads in IP Buffer. Incubate resuspended beads for 5 minutes at 4° with end-over-end rocking.
Repeat the bead washing steps (8.4 through 8.6) 4 more times.

### 9. Denature Sample and Collect from Beads

After all of the immuno-magnetic precipitation tubes have been washed, re-sediment the beads (8.1) and slide tubes into the magnetic holder.
Proteins bound to the immuno-magnetic beads are removed in denaturing conditions. Aspirate off the last IP Buffer wash and remove the IP tube from the magnetic holder.
Add 1x Gel Sample Buffer to the IP tube just above the bead pellet to pool the beads at the bottom of the tube again. The amount of sample buffer depends on the well size of the gel to be used.  You may need to gently tap the bottom of the tube to get all of the beads resuspended in the Gel Sample Buffer.
Repeat the denaturing process (9.2 through 9.3) for each IP tube.
Immuno-magnetic beads resuspended in Gel Sample Buffer are then heated at 75°C for 5 minutes to complete the denaturing process.
Once the sample has been denatured it can be run on an SDS-PAGE gel for western blotting. Emptied Immuno-Magnetic Beads can be re-pelleted by centrifugation and removed from the sample with the magnetic holder.

### 10. Elution of Crosslinked Complexes with Antigenic Peptides (Immunoaffinity Chromatography).

After all of the immuno-magnetic immunoprecipitation tubes have been washed, re-sediment the beads (8.1) and slide tubes into the magnetic holder. Aspirate supernatant and add 10 μl of Buffer A supplemented with the antigenic peptide recognized by the antibody used for immunoisolation of protein complexes. You should test concentrations ranging from 10-200 μM for efficient elution.
Incubate the beads for 2 h at 4°C.
Put beads in the magnetic stand and carefully recover the 10 mL of eluted material. Save this supernatant.
Quickly wash the beads with Buffer A and discard the wash. Save the beads.
Add Gel Sample Buffer to the saved supernatant and beads to a concentration of 1X. Incubate these tubes at 75°C for 5 minutes.
Analyze beads and peptide eluate by SDS-PAGE. The eluate should be free of IgG both by protein stain of SDS-PAGE or by immunoblots (Figure 1B). This material free of IgG is suitable for mass spectrometry.

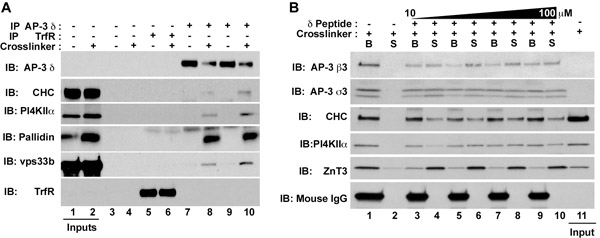
 **Figure 1. Isolation of AP-3 interacting protein complexes and membrane proteins.** HEK293 cells (A) or PC12 cells (B) were treated either in the presence of vehicle control (DMSO, odd lanes Fig 1A) or DSP (even lanes Fig. 1A or all lanes in Fig 1B). Clarified extracts were incubated either with beads alone (Fig. 1A, Lanes 3-4), transferrin receptor antibodies (Fig. 1A, Lanes 5-6), or AP-3 δ antibodies (Fig. 1A, Lanes 7-10; Fig. 1B, lanes 1-10). Immune complexes were eluted with SDS-PAGE sample buffer (Fig. 1A), Buffer A alone or (Fig. 1B, lanes 1-2), or Buffer A supplemented with increasing concentrations of the δ antigenic peptide corresponding to the amino acids 680 710 of human δ-adaptin (NCBI:AAD03777; gi:1923266)(Fig. 1B, lanes 3-10). This peptide binds the δ antibody. After peptide elution, supernatants (S) and beads (B) were analyzed by immunoblot (Fig. 1B). AP-3, which is detected with antibodies against the δ, β3, and σ3 subunits, coprecipitates with the following soluble factors: clathrin heavy chain (CHC), the BLOC-1 subunit pallidin, and the HOPS complex subunit vps33b; as well as the membrane proteins phosphatidylinositol-4-kinase type II alpha  (PI4KIIα) and the zinc transporter 3 (ZnT3). Note the absence of IgG mouse heavy chains in the peptide eluted supernatant in Fig. 1B. 

## Discussion

DSP, a membrane-permeable, chemically reducible crosslinker with a spacer arm of 12 Å is used to stabilize transient protein interactions ^1,2,3,4^. Here we exemplified this strategy with the adaptor complex AP-3 a soluble protein complex that recognizes and sorts membrane proteins into vesicles from endosomes ^5^. AP-3 selectively binds to the zinc transporter ZnT3 and the lipid kinase phosphatidylinositol-4-kinase type II alpha but not transferrin receptor ^1,4,6^. We expanded these observations to the identification of an AP-3 protein interaction network of membrane proteins and cytosolic factors by mass spectrometry ^1^. Low background immuno-magnetic precipitation follows crosslinking to identify interacting proteins. Moreover a published catalogue of proteins that bind non-selectively to magnetic beads allows for a first pass elimination of non-specific protein isolation and identification by mass spectrometry ^7^. DSP utilizes amine-reactive ester groups to link primary amines such as lysines or the amino acid terminus of proteins. Upon denaturing conditions, the DSP molecule is cleaved in half, leaving short chemical groups on the linked amino acids. For some antigens there is a decrease in immunoreactivity signals by immunoblot when comparing equal protein loads between DMSO control and DSP treated cells (Figure1A). It is possible that this decrease results from decreased antibody affinity at the DSP modified lysines. Aside from these rare cases, the use of DSP chemical crosslinking enhances the ability to detect closely interacting membrane-associated proteins.

Proteins or proteins complexes peripherally associated with the cytosolic leaflet of membranes, such as AP-3 are localized to membranes at normal incubation temperatures of 37°C. However, at room temperatures of 15-20°C the AP-3 complex slowly redistributes to the cytosol. Thus, in order to capture interactions between AP-3 and membrane-associated proteins, the internal temperature of these cells must be rapidly cooled to 4°C. The best way to accomplish this is to 1) have all buffers incubating in an ice bath prior to removing cells from the 37°C incubator, 2) to immediately remove all warm media from the plate of cells and replace with ice cold buffer, and 3) to keep the cells suspended on an ice bath for the entirety of the crosslinking experiment.

DSP is not soluble in water and thus warming the PBS/Ca/Mg solution to 37°C before addition of DSP/DMSO solution is advisable. However, since the cells need to maintain a temperature of 4°C, the DSP crosslinking solution should be placed in an ice bath once the DSP is completely solubilized. If the DSP is not completely solubilized large quantities of precipitate will form as the solution cools (a small amount of precipitation is normal). If a large amount of precipitation occurs, try heating the solution back to 37°C for complete solubilization and recool. If the DSP repeatedly falls out of solution, you may need to prepare fresh crosslinking solution. The rapid removal of DSP from solution due to incomplete solubilization should not be confused with the slow formation of a crystalline layer that appears in the wells of DSP treated cells. The presence of this DSP crystalline layer does not interfere with the cross-linking reaction in cells. 
